# Overview of refinement procedures within *REFMAC*5: utilizing data from different sources

**DOI:** 10.1107/S2059798318000979

**Published:** 2018-03-02

**Authors:** Oleg Kovalevskiy, Robert A. Nicholls, Fei Long, Azzurra Carlon, Garib N. Murshudov

**Affiliations:** a Structural Studies Division, MRC Laboratory of Molecular Biology, Francis Crick Avenue, Cambridge Biomedical Campus, Cambridge CB2 0QH, England; b Magnetic Resonance Center (CERM), University of Florence and Interuniversity Consortium for Magnetic Resonance of Metalloproteins (CIRMMP), Via L. Sacconi 6, 50019 Sesto Fiorentino (FI), Italy

**Keywords:** *REFMAC*5, *ProSMART*, *LORESTR*, NMR restraints, refinement

## Abstract

Refinement is a process that involves bringing into agreement the structural model, available prior knowledge and experimental data. To achieve this, the refinement procedure optimizes a posterior conditional probability distribution of model parameters, including atomic coordinates, atomic displacement parameters (*B* factors), scale factors, parameters of the solvent model and twin fractions in the case of twinned crystals, given observed data such as observed amplitudes or intensities of structure factors. A library of chemical restraints is typically used to ensure consistency between the model and the prior knowledge of stereochemistry. If the observation-to-parameter ratio is small, for example when diffraction data only extend to low resolution, the Bayesian framework implemented in *REFMAC*5 uses external restraints to inject additional information extracted from structures of homologous proteins, prior knowledge about secondary-structure formation and even data obtained using different experimental methods, for example NMR. The refinement procedure also generates the ‘best’ weighted electron-density maps, which are useful for further model (re)building. Here, the refinement of macromolecular structures using *REFMAC*5 and related tools distributed as part of the *CCP*4 suite is discussed.

## Abstract 2

Here, a macromolecule-centred approach to three-dimensional structure determination as implemented in *REFMAC*5 is considered. The use of restraints to transfer chemical and structural information during macromolecular refinement, and how different sources of information can be combined in order to achieve models that are more consistent with data derived from a variety of experimental techniques, including macromolecular crystallography, cryo-EM and NMR spectroscopy, are discussed.

## 1. Introduction   

Attempting to understand the three-dimensional structures of macromolecules is akin to opening a ‘black box’: structural models can provide some ideas, or at least hypotheses to test, regarding the function of a molecule of interest. The Protein Data Bank (PDB; Berman *et al.*, 2002 ▸) is the main archive of experimentally determined structural models of macromolecules. The PDB contains models elucidated using three different methods: nuclear magnetic resonance (NMR), X-ray crystallography and electron cryo-microscopy (cryo-EM). Although ultimately all three methods result in an ‘atomic model’, experimental data obtained using these different methods are subjected to different procedures using different tools, reflecting differences in the physical processes underlying each method. Each of these experimental methods produces imperfect data that are insufficient to elucidate a three-dimensional structure with complete certainty. For instance, in an NMR experiment one measures either only short-range interactions (nuclear Overhauser effects; NOEs; Ferella *et al.*, 2012 ▸), distances between the observed nuclei and a paramagnetic metal (pseudocontact shifts; PCSs) or orientations of interatomic vectors connecting two coupled nuclei with respect to an external reference frame (residual dipolar couplings; RDCs) (Koehler & Meiler, 2011 ▸; Bertini *et al.*, 2017 ▸). In the absence of prior structural knowledge, the measured distances and angles may be hard to interpret. In X-ray crystallography, only the intensities of structure factors are measured, so one needs to solve the ‘phase problem’ either by using prior knowledge about a related structure, *i.e.* molecular replacement (Rossmann, 1972 ▸; McCoy *et al.*, 2007 ▸; Vagin & Teplyakov, 2010 ▸), or by carrying out additional experiments and determining phases for a substructure (SAD/MAD, SIR *etc.*; Hendrickson, 1991 ▸; Perutz, 1956 ▸; Sheldrick, 2015 ▸; Skubák & Pannu, 2013 ▸). Often, macromolecules, especially large complexes of exceptional biological interest, fail to form high-quality crystals, resulting in poor diffraction. In such cases, the resulting electron-density maps might be hard to interpret; additional sources of structural information could potentially help to interpret such poor-quality maps. Cryo-EM electrostatic potential maps are the result of averaging thousands of independent but extremely noisy observations, so the local quality (*i.e.* local resolution) of the map varies greatly within the map (Kucukelbir *et al.*, 2014 ▸) and between several reconstructions. Again, the use of additional prior knowledge can help to interpret the low-resolution parts of such maps that exhibit varying signal-to-noise ratios.

In some cases, for example when sufficiently high-resolution X-ray data are available, experimental observations can provide highly accurate information about the position of almost every atom in the ordered part of the crystal; such experimental data may be self-sufficient and not require any additional information in order to successfully build and refine a reliable model. Nevertheless, for the majority of cases, our interpretation of incomplete and noisy experimental observations can be improved by using additional sources of information: the stereochemistry of constituent blocks of macromolecules, typical secondary-structure patterns, structures of related macromolecular domains, structural data obtained using different experimental methods *etc.* (Schröder *et al.*, 2007 ▸; Sheldrick, 2015 ▸; Smart *et al.*, 2012 ▸; Headd *et al.*, 2012 ▸; Nicholls *et al.*, 2012 ▸). Typically, refinement is performed using one model to describe one particular diffraction data set. However, one may have several related diffraction data sets and models, for example with and without the presence of a ligand, or several structures with different ligands. Such data sets may extend to different resolutions and contain data of differing quality. One can even have complementary structural information such as PCSs and RDCs from NMR experiments (Koehler & Meiler, 2011 ▸; Bertini *et al.*, 2017 ▸). Attempts have been made to use data obtained in biochemical protein–protein interaction studies for structural modelling (Förster & Villa, 2010 ▸), and SAXS data may also provide complementary information (Shevchuk & Hub, 2017 ▸). We would like to be able to address important biological questions by simultaneously co-utilizing all the available structural information. Ideally, all the available sources of experimental and theoretical information relevant to the molecule of interest should be integrated into one process, with the intention of delivering the best possible structural model for a given state of the molecule.

From a practical point of view, a reliable mathematical framework is required in order to integrate various different sources of information. The problem of macromolecular structure refinement against experimental data can be viewed as a statistical problem whereby some prior knowledge about the system under study is available. Bayes’ theorem indicates that whenever we obtain any additional evidence, our estimate of the probability of a particular state changes from some prior estimate to a different posterior (Kendall *et al.*, 1994 ▸). Therefore, the Bayesian framework is perfectly suited to integrating various and heterogeneous sources of information into a single workflow (Bricogne, 1997 ▸), using approximations, where necessary, to speed up calculations. Modern refinement programs use the maximum-likelihood method in order to estimate model parameters, whilst ensuring good agreement with both prior knowledge and experimental data (Bricogne & Irwin, 1996 ▸; Pannu & Read, 1996 ▸; Adams *et al.*, 1997 ▸; Murshudov *et al.*, 1997 ▸). Refinement programs consider the merged intensities of structure factors and their standard deviations as experimental observations.

One longstanding problem in the field is that of refinement against unmerged intensities, or even diffraction images. Such a refinement procedure could allow the estimation of changes in the crystal during data collection, for instance, to take into account the effects of radiation damage on the structure. Although some effort has been made to implement such a refinement procedure (Fancher *et al.*, 2016 ▸), there is no universal satisfactory solution as of yet.

Note that experimental observations are typically incomplete and noisy, for example when the resolution of X-ray or cryo-EM data is insufficiently high, or when only a subset of the interatomic distances is registered during the course of an NMR experiment. Therefore, there will typically be many potential models that are consistent with the same set of observations. One approach intended to resolve this issue is the refinement of model ensembles (Burnley *et al.*, 2012 ▸; Phillips & Cole, 2012 ▸; Hummer & Köfinger, 2016 ▸). Another approach is to use additional information (prior knowledge) to choose one model; the maximum *a posterior* probability (MAP) estimation method serves this purpose. As the name suggests, this technique maximizes the posterior conditional probability of the model parameters given the current observations (Murshudov *et al.*, 2011 ▸). Further to the experimental data suffering from noise and incompleteness, the model provided for refinement could be incomplete, with missing parts ranging from small-molecule ligands to whole domains at the early building stages. Missing parts create specific problems with scaling and solvent modelling, as many refinement programs would consider unmodelled parts as a ‘solvent’ region. One of the possible solutions to this problem is to consider the macromolecular envelope as a continuous distribution, not a binary mask (Roversi *et al.*, 2000 ▸).

Crystal diffraction and cryo-EM data rarely contain information about bond lengths or angles; only data beyond 1.2 Å resolution can contain sufficient information to allow accurate estimation of the distances between well defined atoms (Sheldrick, 2015 ▸). Consequently, the prior probability distribution must minimally contain information about bond lengths and angles. As the resolution decreases, longer and longer range information, such as torsion angles and information relating to secondary structures and the composition of domains, might be needed. This reflects a general principle: when complementing data with prior knowledge that is not contained in the data, the less experimental evidence one has the more one must rely on prior knowledge, thus the more prone refinement is to suffer bias towards prior information. An important consequence of this is that in order to address biological questions, one needs experimental data that extend to a resolution high enough to elucidate a reliable model at the required level of detail. For instance, low-resolution data are typically sufficient to determine the general arrangement of subunits in a large macromolecular complex, but atomic resolution data are required in order to clarify the finer details of catalysis.

In this paper, we review the Bayesian framework as implemented in the macromolecular structure-refinement program *REFMAC*5 (Murshudov *et al.*, 2011 ▸), noting that comparable technologies are employed by other refinement software. We will focus on refinement against crystallographic diffraction data, but the same principles are applicable to refinement against cryo-EM maps (Murshudov, 2016 ▸; Brown *et al.*, 2015 ▸). We will discuss how the formalism of restraints (chemical restraints and external restraints) can be used to incorporate prior knowledge (including information obtained using different experimental methods) into the refinement process, and even establish information flow between different but related structures.

## 2. Resolvability of peaks   

**Figure 1:**
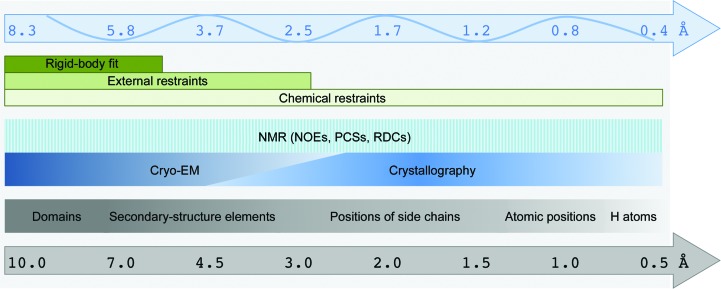
Experimental data and complementary prior knowledge *versus* resolution. The grey arrow shows resolution in Å and the grey bar above it indicates the level of macromolecular structural detail that can be observed in the corresponding resolution range. The blue panel shows experimental methods that deliver information in the corresponding resolution range; the NMR bar is coloured with a pattern to reflect the different nature of NMR-derived data (distances between points rather than a continuous distribution of density). The green panel shows structural restraints that are useful for complementing the experimental data that are missing at certain resolutions. The top arrow indicates the minimal theoretical resolvability of peaks in the absence of noise at a given resolution.

It is necessary to analyse the information contained in the data, and to use enough prior knowledge to complement this information. Both crystallographic and cryo-EM techniques produce data pertaining to long-range interactions between atoms in the molecule; shorter range interactions become available as the resolution of the data increases (Fig. 1 ▸). There are several techniques to define the optical resolution of data (Vaguine *et al.*, 1999 ▸; Urzhumtseva *et al.*, 2013 ▸). Since the quoted resolution of experimental data is determined in reciprocal space, it is interesting to estimate the minimal distance between two points in real space that can be resolved using diffraction data of a certain resolution. It is easier to estimate the minimal theoretically resolvable distance between two points if we assume the absence of noise and zero *B* factors, *i.e.* under the assumption that the data are ideal. Here, we consider the Fourier transformation of a sphere with radius *s*_max_ to demonstrate how a single peak is broadened when only data extending to limited resolutions are available (see, for example, Pinsky, 2001 ▸), 
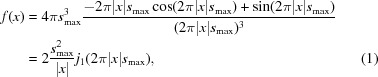
where *j*_1_ is a spherical Bessel function of order 1.

We can distinguish two peaks at a distance |*x*| if the following criterion is satisfied,
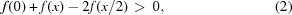

*i.e.* there is less density in the middle of two neighbouring peaks than at the top of those peaks. The equation *f*(0) + *f*(*x*) − 2*f*(*x*/2) = 0 has the solution at |*x*|*s*_max_ ≃ 0.8322, meaning that, for a given resolution *s*_max_ = 1/*d*_max_, the minimal interatomic distance that can be distinguished in the density is |*x*| ≃ 0.8322/*s*_max_ = 0.8322*d*_max_. We call this the maximum possible resolvability of peaks for a given diffraction data set with resolution *d*_max_. In practice this limit is never achieved. Obviously if the data are not perfect, for example, owing to incompleteness, noise in the data or atomic mobility expressed as *B* factors, then the resolvability will be reduced accordingly. Moreover, resolvability in different directions could be expected to be different and, since the mobility of atoms in different parts of the molecule could be different, we would expect resolvability to also depend on position. In the case of perfect data, if the resolution is 5 Å then the resolvability of peaks is around 4.16 Å. This means that to derive accurate atomic models using diffraction data extending to 5 Å resolution we need prior information about interatomic distances to at least 4.16 Å.

## 3. Restraints: universal formalism for information transfer   

### 3.1. Chemical restraints: implementation   

During refinement against X-ray data, *REFMAC*5 minimizes a target function that has two components:
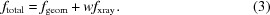

*f*_geom_ is the contribution of the geometry term (the negative log prior probability distribution, representing our prior chemical and structural knowledge), *f*_xray_ is the contribution from the experimentally observed data (the negative log-likelihood function, representing the probability of the data given the current model) and *w* is a weight specifying the relative contribution of these terms.

As part of the refinement process, we need to estimate and improve the quality of the model. As such, owing to the fact that we aim to optimize the probability of the model during refinement, structures with reasonable stereochemistry (*i.e.* those containing fewer Ramachandran/bond-length/angle outliers, and thus that are considered to be more energetically favourable) should be more abundant in the PDB than those with poor stereochemistry. Indeed, we aim to improve the quality of the model by improving its consistency with our prior knowledge about stereochemistry, provided that enforcing such an agreement does not contradict experimental observations. This knowledge is applied in the form of so-called restraints. Chemical restraints encapsulate chemical information about the constituent blocks (*e.g.* amino acids, nucleic acids, ligands) of macromolecules and the covalent links between them. Standard restraints between covalently linked atoms have the general form
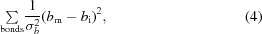
where *b*_m_ represents a geometric parameter (*e.g.* bonds, angles, chiralities) calculated from the current model and *b*_i_ is the ideal value of this particular parameter from a pre-tabulated dictionary (Murshudov *et al.*, 2011 ▸). Correspondingly, the efficiency of refinement depends on the quality of the dictionary it uses. Originally, *REFMAC*5 used a dictionary built by *LIBCHECK* (Vagin & Teplyakov, 1998 ▸).

### 3.2. Obtaining stereochemical restraints to use as prior knowledge   

There are several programs that can be used to derive stereochemical information for a given chemical compound using information about the bonding pattern. These include *phenix.elbow* (Moriarty *et al.*, 2009 ▸), *grade* (Smart *et al.*, 2012 ▸), *ProDRG* (Schüttelkopf & van Aalten, 2004 ▸) and *PURY* (Andrejašič *et al.*, 2008 ▸). One of the programs distributed as part of the *CCP*4 suite (Winn *et al.*, 2011 ▸) is *AceDRG* (Long *et al.*, 2017 ▸). *AceDRG* uses the Crystallography Open Database (COD; Gražulis *et al.*, 2009 ▸), which is a freely available small-molecule database, to extract, validate and tabulate bond lengths and angles for various pairs and triplets of atom types that are based on the local chemical neighbourhood of atoms (Long *et al.*, 2017 ▸). *AceDRG* is the prime source of chemical prior knowledge used by *REFMAC*5, although if a user wishes they can use their own library of stereochemical information generated by other available programs. Further details of *AceDRG* can be found in Long *et al.* (2017 ▸).

### 3.3. Other types of restraints   

Noncrystallographic symmetry (NCS) restraints are an interesting special case of restraints. They are applicable in cases where several monomers of the same protein are observed in the asymmetric unit (Jones & Liljas, 1984 ▸; Murshudov *et al.*, 2011 ▸). Chemically identical protein monomers usually adopt very similar conformations, but may differ in small details such as flexible-loop positions. *REFMAC*5 can utilize this in several ways: by applying NCS constraints (all NCS-related copies are considered to be exactly the same, so only one set of atomic parameters per molecule is refined), global NCS restraints (molecules are superposed and the differences between atomic positions are minimized) or local NCS restraints (differences between local interatomic distances are restrained) (Murshudov *et al.*, 2011 ▸). Using NCS restraints, rather than refining near-identical structural regions independently, reduces the effective number of refined parameters of the model and thus improves the observation-to-parameter ratio.

Generally, the ratio between the number of observations and the number of adjustable model parameters is very small at low resolution, leading to unstable refinement with the danger of overfitting. To address this problem, the so-called ‘jelly-body’ restraints were introduced specifically to stabilize refinement at early stages and at low resolution (for example below 3.0 Å). Jelly-body restraints use the current interatomic distances in the model as the target (Murshudov *et al.*, 2011 ▸), thus restraining the independent movement of atoms within a certain radius, leading to concerted motion. By their nature, jelly-body restraints do not change the value of the likelihood function. Rather, they affect only the second derivative of the likelihood function, changing the search landscape and improving convergence. We recommend using this option immediately after molecular replacement, for resolutions below 3.0 Å or whenever a large radius of convergence is needed.

## 4. External restraints   

### 4.1. External restraints: implementation   

Secondary and tertiary structures of macromolecules are maintained by noncovalent interactions: hydrophobic, van der Waals, hydrogen-bond and electrostatic interactions (salt bridges). Despite having a different physical nature, these interactions effectively hold atoms together/apart at certain distances, allowing protein or nucleic acid chains to form higher order structures. Fortunately, we are able to use the formalism of restraints to describe these noncovalent contacts (Nicholls *et al.*, 2014 ▸; Headd *et al.*, 2012 ▸; Smart *et al.*, 2012 ▸) *via* the so-called external restraints. Target values for these restraints can be derived not only by considering idealized secondary-structure elements and hydrogen-bonding patterns, but also from any available homologous structures. Indeed, external restraints can be a powerful mechanism for information transfer, being used to inject prior knowledge into the refinement process. If external restraints are applied, the target structure is pulled towards the conformation adopted by the reference structure(s) or idealized structural fragment conformations.

The use of external restraints to homologous structures involves applying restraints on the distances between proximal atom pairs based on a presumed atomic correspondence between the two homologous structures. The following function is used for external structure restraints,
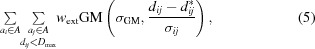
where the atoms *a*_*i*_ belong to the set *A* of atoms for which a correspondence between target and reference structures is known, *d*_*ij*_ is the distance between the positions of atoms *a*_*i*_ and *a*_*j*_, 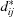 is the corresponding distance in the known homologous structure, σ_*ij*_ is the estimated standard deviation of *d*_*ij*_ about 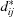 and *D*_max_ ensures that atom pairs are only restrained within localized regions, so as to allow global conformational changes (Nicholls *et al.*, 2012 ▸). External restraints should be weighted differently to the other geometry components in order to allow the restraint strength to be separately specified. Consequently, a weight *w*_ext_ is applied, which should be appropriately chosen depending on the data quality and resolution, the structural similarity between the external known structure and the target, and the choice of *D*_max_. The Geman–McClure (GM) robust estimation function,
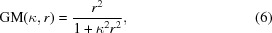
is used to increase robustness (decrease sensitivity) to outliers (Huber, 1981 ▸), where *r* is typically the normalized difference between two values (target and observed values) and κ is a sensitivity parameter.

Effectively, external restraints can stabilize refinement in difficult cases where the information content of the diffraction data is low and, consequently, the effective observation-to-parameter ratio is poor. Introducing external restraints reduces the effective number of adjustable parameters and therefore reduces overfitting of the model into the noise. It also changes the landscape of the function to be minimized, thus increasing the radius of convergence of refinement (*i.e.* in the case where the model is currently in an incorrect conformation, external restraints can help the model to escape local minima of the target function by pulling it into the correct conformation).

### 4.2. *ProSMART*   

The program *ProSMART* (Nicholls *et al.*, 2014 ▸) works in tandem with *REFMAC*5 by supplying restraints on local interatomic distances using known structural models of homologous proteins or nucleic acids, backbone hydrogen bonds detected in the target structure, or backbone conformations corresponding to secondary-structure elements. Specifically, *ProSMART* analyses the structural similarity between the target and reference models before identifying an atomic correspondence, distances 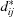 and standard deviations σ_*ij*_. *ProSMART* then generates corresponding local inter­atomic distance restraints for atom pairs that are sufficiently spatially proximal, as defined by a distance threshold *D*_max_ (see equation 5 above).

**Figure 2:**
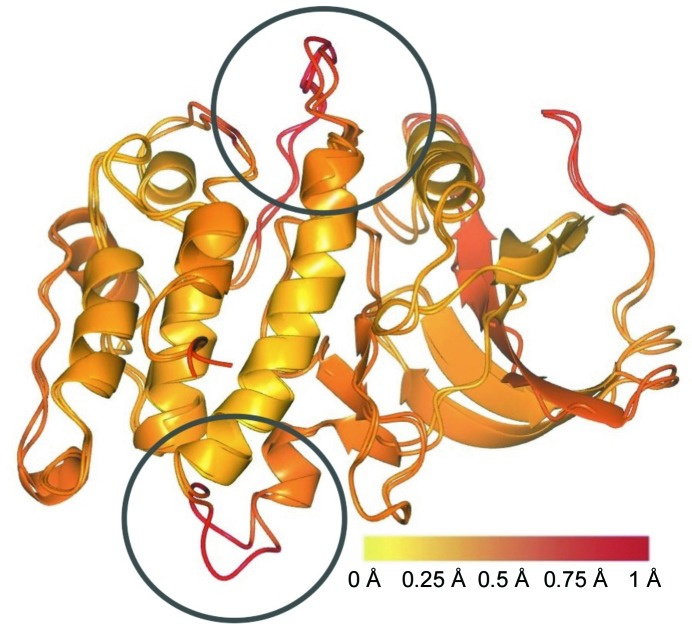
Automated refinement of PDB entry 1jkt with the *LORESTR* pipeline and comparative structural analysis using *ProSMART*. The figure shows superposition of the model with PDB entry 1jkt before and after automated re-refinement (the corresponding refinement statistics are shown in Table 1 ▸). Both structures are coloured according to local backbone deviation using *ProSMART* (as described by Nicholls *et al.*, 2014 ▸). This representation allows quick and easy visual identification of exactly which regions have changed during re-refinement. The colour gradient indicates local backbone r.m.s. deviation in the vicinity of each residue; residues coloured yellow retain their conformation, whilst those coloured red exhibit substantial structural changes during refinement. Two regions that have undergone dramatic local structural changes are highlighted by grey ovals. In practice, the electron-density maps in such regions would be manually inspected in order to assess the reasons for such changes to the model and to determine how best to proceed with further model building and refinement. The figure was prepared using *CCP*4*mg* (McNicholas *et al.*, 2011 ▸).

The core functionality of *ProSMART* is to provide a structure-based alignment and to perform a detailed local structural comparison (see Fig. 2 ▸), particularly in the presence of global conformational changes such as domain motion (and without requiring reliable or even similar secondary/tertiary/quaternary-structure organization). Indeed, in the context of restraint generation and information transfer, it is not important for the global organization of the homologous model and the structure under refinement to be the same; the reference and target structures are not required to superpose well in order to achieve success with external restraints. This is important for external restraint generation in cases where a high-resolution structure may have been solved in a different conformational state; if local structure is conserved then this information can be exploited during refinement. For this reason, the approach to alignment implemented in *Pro­SMART* involves searching target and reference models for regions that are highly conserved in terms of local backbone structure, not necessarily in terms of the overall organization at the domain level. Note that this flexible approach, which is independent of global conformation, contrasts with the many conventional structural alignment programs that instead aim to optimize a superposition. However, in cases where the relative organization of domains varies between target and reference structures, any rigid subdomains for which local structure is highly conserved will be subsequently identified by *ProSMART* for further analysis.

For external restraints based on homologous structures, *ProSMART* performs local structural alignment of the target and reference chains, identifying matching atoms. Then, for every atom in the reference chain that matches an atom in the target chain, the program searches within a particular distance (the default value is 4.2 Å) for proximal atoms that are not covalently bound. The default value of 4.2 Å was estimated by extensive tests; this value is large enough to pick up non­covalently bound atom pairs within and between secondary-structure elements, but small enough to avoid enforcing global rigidity, thus allowing large-scale conformational changes such as domain movement. After identifying the list of appropriate atom pairs, *ProSMART* records the interatomic distances found in the reference structure(s) as the objective values of the restraints, which are subsequently used by *REFMAC*5 during refinement of the target structure. In order to use such external restraints, one or more reference structures, sufficiently similar to the target, must be available. *ProSMART* is also capable of dealing with multiple structures; in this case it will record all identified distances between the same pair of atoms in all homologous structures. *REFMAC*5 inspects these distances and chooses the closest value to that observed in the current state of the target structure as the objective value of the restraint.

In the case of hydrogen-bond restraints, *ProSMART* detects potential hydrogen bonds (the detection range is 2–3.5 Å) between main-chain atoms in the target structure under refinement (no reference structures are needed) and uses a standard hydrogen-bond length (2.8 Å) as the objective value in the current implementation. Hydrogen bonds, being an electrostatic interaction, may vary in length from 2.2 to ∼4 Å (Jeffrey, 1997 ▸); using a fixed target length of 2.8 Å (this is the average N—H⋯O hydrogen-bond length derived from crystals of small peptides; Pauling, 1960 ▸) proved to work well in our tests. These restraints help to maintain structural integrity of the main-chain conformation at low resolution (3–5 Å). *ProSMART* can also generate restraints based on standard conformations: it detects α-helical and β-strand-like fragments in the target structure, and the distances found in the reference ideal structures are then used as the objective values of the restraints.

One of the dangers with this approach occurs when the high-resolution homologous model used for restraint generation does not closely represent the true structure underlying the low-resolution model under refinement. This can occur when the homologous model contains errors, or when there are true local differences between the two structures. The use of the Geman–McClure robust estimation function during refinement, as described above, helps to avoid the negative consequences of this, to some degree, by naturally downweighting any restraints corresponding to extreme inconsistencies between the target and reference models.

Since it would be undesirable for destructive information to be transferred from poor-quality homologous models to low-resolution models during refinement, it is recommended to inspect and exclude any obviously poor regions of the homologous models from being used during external restraint generation. *ProSMART* attempts to automatically detect potentially unreliable regions in the homologous model by inspection of the distribution of atomic *B* factors; atoms with *B* factors much larger than those of the vast majority of the atoms in the model (higher than the median plus twice the interquartile range) are excluded from restraint generation.

However, there is no substitute for manual user due diligence. Indeed, it is recommended to actively consider the quality/reliability of any homologous models before using them for restraint generation. One resource that is particularly useful for this purpose is *PDB_REDO* (Joosten *et al.*, 2014 ▸), which contains re-refined (and in some cases rebuilt) versions of models taken from the PDB, as well as providing annotation on their quality.

### 4.3. *LIBG*   

*LIBG* (Brown *et al.*, 2015 ▸) is another program that can generate restraints for use by *REFMAC*5; it produces restraints specifically to maintain nucleic acid geometry during the refinement process. *LIBG* generates restraints for canonical Watson–Crick and noncanonical G:U base pairs; it also generates restraints to preserve stacking interactions between nucleic acid bases and planar side chains of protein amino acids (parallel-plane restraints).

Putative base pairs are identified by inspecting the local neighbourhood around the N and O atoms of a base for hydrogen-bond candidates in an adjacent base. A base pair is selected if the combination of hydrogen-bonding patterns between two bases satisfies the preset patterns of hydrogen bonding between DNA/RNA base pairs, and the values of the hydrogen-bonding lengths, torsion angles and features of chirality are within the allowed deviation ranges from the corresponding reference values, which are estimated statistically from a database of high-resolution X-ray and neutron crystal structural models (Xin & Olson, 2009 ▸). Users can adjust these criteria by changing the allowed deviations.

For stacking interaction restraints, possible pairs of stacking planes are determined by calculating the angle between the normals of two planes in different DNA/RNA bases or protein amino acids, the angles between the normal of one plane and the vector linking the two ‘gravity’ centres of planar atoms, and the distance between those two ‘gravity’ centres. If the calculated values are within predefined ranges then the two planes are selected as candidates for stacking. The selected pairs are used by *REFMAC*5 to make sure that they stay parallel during refinement.

### 4.4. *Low-Resolution Structure Refinement* pipeline (*LORESTR*)   

*ProSMART* has proven to be a useful tool for aiding the refinement of difficult cases at low resolution. However, many decisions (the selection of homologues for restraint generation, choosing optimal modes and parameters for both *Pro­SMART* and *REFMAC*5) are left to the user, and obtaining the best possible results requires substantial manual effort and optimization of parameters through trial and error. We have recently tested various refinement strategies and different *REFMAC*5 and *ProSMART* parameters on a test set of more than 100 structures with resolution below 3.0 Å taken from the PDB. The best-performing refinement protocols and strategies have been implemented in *LORESTR*, an automated and easy-to-use pipeline for structure refinement at low resolution, which is distributed as part of the *CCP*4 suite v.7.0 (Kovalevskiy *et al.*, 2016 ▸; Winn *et al.*, 2011 ▸).

The minimal input required by *LORESTR* is a PDB file containing the current model (the target structure) and an MTZ file containing the corresponding diffraction data. In automatic mode, it extracts the sequences of all chains present in the PDB file and runs a *BLAST* search (Altschul *et al.*, 1997 ▸) over the whole PDB (an internet connection is required). It then downloads all homologues that share at least 75% sequence identity and cover at least 75% of the protein chain (these values were identified during extensive testing using models from the PDB). *LORESTR* specifies hydrogen-bond restraints to be used by *ProSMART* for any chains for which no close homologues are found. Users can also manually supply any number of homologous structures (PDB files). This is useful, for instance, in cases where the PDB files are private and/or not yet released in the PDB.

After downloading homologues, the pipeline analyses the input data in order to determine the set of most appropriate refinement parameters (scaling method, solvent parameters and twinning). After analysing and ranking the available homologous chains for restraint generation, the pipeline generates a number of refinement protocols, depending on the number of available homologous chains. If no homologues are supplied and no homologues are found during the *BLAST* search, the pipeline will just test the two protocols that do not require the availability of external homologues, *i.e.* hydrogen-bond restraints and jelly-body restraints (Murshudov *et al.*, 2011 ▸). For all protocols for which external homologues are available, the pipeline runs one round of *REFMAC*5 refinement using external restraints, before then executing a second round of refinement using only jelly-body restraints in order to allow the structure to relax into its new conformation (as this approach proved to be optimal in the vast majority of test cases). The pipeline supports multitasking and can run several jobs in parallel, should the user so wish. After running all jobs, *LORESTR* selects the best-performing protocol according to a quality indicator (Kovalevskiy *et al.*, 2016 ▸) that depends on both *R*_free_ and the *MolProbity* score (Chen *et al.*, 2010 ▸), or just simply *R*_free_ if *MolProbity* is not available from a local *PHENIX* installation (Adams *et al.*, 2010 ▸). The refined PDB and MTZ files corresponding to the best protocol are returned.

*LORESTR* has a mode specifically designed for automated refinement directly after molecular replacement. In this case, before running the set of standard refinement protocols the pipeline runs 100–200 cycles (depending on the starting *R* factors) of refinement using jelly-body restraints in order to relax the structure into its new position.

In our tests, *LORESTR* was able to produce substantially better quality models in the vast majority of cases, improving both the *R* factors and the model geometry for 94% of the test cases (Kovalevskiy *et al.*, 2016 ▸). The dramatic improvement in *R* factors and stereochemical quality of low-resolution models observed when using the fully automated mode of the pipeline demonstrates its potential utility in low-resolution cases, especially during the initial stages of refinement, or when the refinement process has stalled.

### 4.5. Example of automated re-refinement using *LORESTR*   

To illustrate the potential of *LORESTR*, we used it to re-refine the 3.5 Å resolution model of death-associated protein kinase with PDB code 1jkt (Tereshko *et al.*, 2001 ▸), which comprises two protein chains. *LORESTR* was used to automatically optimize the refinement protocol and re-refine the model using *REFMAC*5, aided by external restraints generated by *ProSMART*. It was automatically determined that the data were twinned (two domains with fractions refined to 66 and 34%). The optimal *LORESTR* protocol involved 40 cycles of refinement using external restraints generated from a combination of four of the homologous structures available in the PDB (2x0g chain *A*, 2xuu chain *A*, 4b4l chain *A* and 4tl0 chain *A*), followed by a further 20 cycles of refinement using jelly-body restraints (without any external restraints).

Refinement and geometry statistics corresponding to the original and re-refined model are provided in Table 1 ▸. Both *R*_work_ and *R*_free_ were dramatically reduced, indicating a much better fit of the model to the data after refinement. Furthermore, all geometry statistics were improved, implying the model to be more consistent overall with the prior chemical and structural knowledge. Fig. 2 ▸ illustrates local structural differences between the original model 1jkt that was deposited in the PDB and the model that arises from automatic re-refinement using *LORESTR*.

The optimal refinement protocol from *LORESTR* resulted in substantial reductions in both *R* and *R*_free_, which both decreased by over 7%. Importantly, the ∼4% difference between the *R* factors was maintained (Δ*R* decreased from 4.1 to 3.9%) indicating stable refinement without excessive overfitting. We see that the use of external restraints results in greatly improved backbone geometry, indicating that a more reasonable model [as can be seen by inspecting the *MolProbity* score percentile (Chen *et al.*, 2010 ▸) as well as the Ramachandran statistics before and after refinement; see Table 1 ▸]. Note that backbone torsion angles are not explicitly restrained by the external restraints. Rather, the general improvements in their values are a consequence of the stabil­ization of local structure, which is achieved in interatomic distance space.

### 4.6. Information flow for the refinement of two related low-resolution structures   

It is clear that low-resolution structure refinement often benefits from restraints based on high-resolution homologues. We can consider another question: can we improve the refinement of several related low-resolution structures by information transfer between them, assuming that no high-resolution homologues are available? It is possible to use the formalism of restraints to pass information between such structures in a hope that the information flow will cooperatively lead to improvements in both models. The implied refinement strategy is simple: generate external restraints using one structure and use them to refine the other, then generate restraints using the second structure and use them to refine the first. This cycle could be repeated until convergence (*i.e.* the *R*_free_ does not further decrease during refinement).

We have tested this strategy on 28 pairs of low-resolution structures from the PDB, testing not only protocols with external restraints, but also using simple jelly-body and hydrogen-bond restraints. For 15 of the pairs this refinement strategy resulted in improvements in *R*_free_ for both structures in the pair, in ten of the pairs *R*_free_ was better for one of the structures but worse for the other, and for three cases this procedure failed to improve the fit of either model to the diffraction data. The geometric quality, as judged by the *MolProbity* score percentile (Chen *et al.*, 2010 ▸), was improved for the vast majority of successful cases (Supplementary Table S1). On average, three macrocycles of iterative refinement of the two models were sufficient to achieve convergence.

The relatively low success rate, compared with refinement with restraints generated based on high-resolution homologues, could be explained by the suboptimal geometric quality of the starting low-resolution structures. This would cause the iterative cooperative refinement to suffer from error propagation, rather than to benefit from the positive effects of information transfer. We have tried to improve performance by the automated removal of restraints from imperfect parts of the models (residues with either a *B* factor higher than the median plus interquartile range or a real-space correlation below the median minus interquartile range). Applying this procedure resulted in further improvement of *R* factors and geometrical quality for more than half of the test structures, but the overall behaviour remained the same (see Supplementary Table S1). Only one structure that could not be improved using restraints generated for the whole model was improved after the removal of restraints for imperfect parts. This may indicate that the current implementation of external restraints in *ProSMART* and *REFMAC*5 is robust enough towards incorrect distances that such simple procedures as the removal of restraints corresponding to bad parts of the model do not improve refinement dramatically. However, refinement being improved for more than half of the low-resolution test pairs suggests that this approach could be useful for some cases. Strategies for the cooperative refinement of multiple low-resolution structures require further investigation.

Note that there have been previous attempts to use a Bayesian approach in order to co-utilize information from multiple structures in other contexts, for example in the refinement of differences between isomorphous structures (Terwilliger & Berendzen, 1996 ▸).

## 5. NMR restraints in refinement   

**Figure 3:**
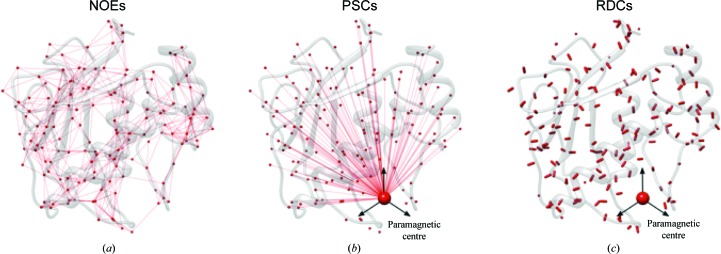
NMR-based structural restraints commonly used in model refinement. (*a*) NOEs provide short-range interatomic distances, (*b*) PCSs give long-range distance and angular information of the metal–nucleus vectors relative to an external reference frame, and (*c*) RDCs provide angular information corresponding to vectors connecting two nuclei relative to the external reference frame. In cases where the alignment of the molecule arises from the presence of a paramagnetic centre, PCS and RDC restraints have the same reference frame.

As opposed to X-ray crystallography, NMR spectroscopy is limited in terms of the amount of data that can be obtained. Typical NMR experiments deliver torsion-angle restraints (from chemical shifts) and short-range distances (<10 Å) that are obtained by dipolar cross-relaxation (nuclear Overhauser effect; NOE; Ferella *et al.*, 2012 ▸). It is a computationally challenging task to deconvolute a set of distances and angles into a three-dimensional macromolecular structure or even complex. Thus, it is a rather long and inefficient task to obtain a high-resolution structure by NMR spectroscopy. However, NMR-based restraints measured in solution can make a valuable contribution to the structural refinement of macromolecules against diffraction data. Two kinds of restraints are particularly useful in this sense: PCSs and RDCs (Fig. 3 ▸). The first can be measured to cover distances spanning the whole macromolecule, whereas the latter yield highly accurate angle measurements. These restraints have thus played a fundamental role in the development of structure-determination/refinement strategies by solution NMR spectroscopy, and their use has become increasingly popular in recent years (Koehler & Meiler, 2011 ▸). PCSs and RDCs owe their popularity to the fact that, in contrast to other classical NMR parameters, these restraints allow the extraction of distance and angular information relative to an external reference frame, making it possible to obtain information about the overall molecular system.

Pseudocontact shifts, which give ‘long-range’ restraints, originate from the presence of a paramagnetic metal in the molecule. The corresponding electron spin gives rise to an average magnetic moment (the Curie spin) that may have different magnitudes for different orientations of the molecule with respect to the applied magnetic field. All of the NMR-active nuclei in the molecule have a dipolar interaction with the Curie spin and, if the Curie spin is anisotropic, the nuclei sense the dipolar interaction with it as a distortion of the external magnetic field, which is described by a law taking the form (Bertini *et al.*, 2002 ▸)
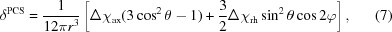
where Δχ_ax_ and Δχ_rh_ are the axial and rhombic components of the magnetic susceptibility anisotropy tensor **χ**, *r* is the distance between the nucleus and the paramagnetic centre, and θ and φ are the spherical angles describing the orientation of the metal–nucleus vector with respect to the principal axes of the **χ** tensor. PCSs depend on the distance between the observed nucleus and the paramagnetic metal as 1/*r* ^3^ and are usually measurable over ∼40 Å.

RDCs arise when the internuclear dipolar interaction is not averaged to zero upon rotation, which happens if not all of the orientations are equally possible for the molecule. RDCs give information on the orientation of interatomic vectors connecting two coupled nuclei with respect to an external reference frame, providing ‘global’ information about the molecular system, which nicely complements other NMR restraints that have a more ‘local’ character (*i.e.* NOEs). The situation of partial alignment may be encountered in anisotropic media such as liquid crystals (Otting *et al.*, 2000 ▸), upon interaction of the molecule with aligned objects such as bicelles (Sanders & Landis, 1995 ▸) or filamentous phages (Bax, 2003 ▸), and occurs when there is self-alignment of the molecule in the field (Tolman *et al.*, 1995 ▸). Self-alignment originates from anisotropy of the magnetic susceptibility, and is larger in the case of paramagnetic systems. In this case, RDCs are linked to the same tensor quantity that describes the PCSs. Hence, the external reference frame can be assumed to be common and the restraints can be used profitably together. RDCs are described by the following equation (Bertini *et al.*, 2002 ▸),
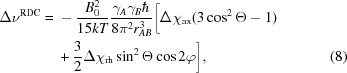
where Δχ_ax_ and Δχ_rh_ are the same as in (6), *r*_*AB*_ is the distance between the nuclei *A* and *B*, Θ and Φ are the spherical angles describing the orientation of the interatomic vector connecting nuclei *A* and *B* with respect to the principal axes of the χ tensor, *B*_0_ is the magnetic field, γ_*A*_ and γ_*B*_ are the gyromagnetic ratios of nuclei *A* and *B*, and 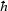 = *h*/2π, where *h* is Planck’s constant.

PCSs and RDCs not only provide different kinds of information about the geometry of the molecule, but PCSs are also less sensitive to small local structural inaccuracies and thus are used to provide a more robust estimation of the paramagnetic tensor to which the external reference frame refers to, while RDCs are very sensitive to small conformational rearrangements and thus are used as powerful probes for the detection of structural disorder and/or molecular motions on timescales of up to milliseconds (Ulmer *et al.*, 2003 ▸; Clore & Schwieters, 2004 ▸; Lindorff-Larsen *et al.*, 2005 ▸; Lange *et al.*, 2008 ▸). To experimentally measure RDCs and PCSs, paramagnetic metals can be introduced either by substitution of a diamagnetic metal, if originally present in the molecule, or by the attachment of rigid tags coordinating a paramagnetic metal (Ma & Opella, 2000 ▸; Franz *et al.*, 2003 ▸; Keizers *et al.*, 2007 ▸; Otting, 2008 ▸; Su *et al.*, 2008 ▸; Häussinger *et al.*, 2009 ▸; Saio *et al.*, 2009 ▸).

Owing to their ‘global’ character, PCSs and RDCs represent the best candidates for shedding light on the longstanding issue of the combination of structural knowledge derived from solution and solid-state (crystal) data (Carlon, Ravera, Andrałojć *et al.*, 2016 ▸). NMR data, indeed, are often used in comparison (or, ideally, in combination) with crystal diffraction data, whenever available. Also, crystal data have been used to improve solution NMR structures (Brunner *et al.*, 2006 ▸). Interestingly, NMR data frequently show limited compatibility with models obtained from crystallographic data. Such discrepancies may either reflect real differences between the true structures of the molecules as found in the crystalline and the solution state, or may be owing to imprecision of the atomic positions determined in the crystal diffraction experiment, when the resolution of the data is not atomic. NMR restraints can be used not only to validate the crystal model but also potentially to improve it by directly including solution data in the refinement process.

*REFMAC*5 (Murshudov *et al.*, 2011 ▸) has been extended to allow the inclusion of PCSs and RDCs as additional restraints during the refinement process (Rinaldelli *et al.*, 2014 ▸). The main purposes are to (i) produce a model that is compatible with both experimental methods and (ii) identify any genuine differences between the solution and crystal state that lie outside the range of experimental uncertainty. This refinement protocol is best applied to medium-to-low resolution structure refinement. The presence of a resolution-dependent inaccuracy in the atomic coordinates, the ‘structural noise’, has been proven to significantly impact the agreement between solution data and the X-ray-derived model, affecting the interpretation of the data and limiting the extraction of structural and dynamic properties. The positions of H atoms are not accessible by X-ray crystallography unless one is lucky enough to obtain a crystal that diffracts to ultrahigh resolution. However, H atoms are the major source of information for NMR spectroscopy. Localization of the H atoms in the crystal structure is usually based on the position of heavy atoms according to ideal covalent geometries (‘riding H atoms’; Wlodawer *et al.*, 2008 ▸; Word *et al.*, 1999 ▸). A minor inaccuracy in the orientation of the heavy atoms is then translated into relevant inaccuracies in the placement of the H atoms (Zweckstetter & Bax, 2002 ▸).

### 5.1. Refinement against diffraction data using NMR restraints   

Practical refinement with *REFMAC*5 using NMR restraints involves adding the contribution of PCSs and/or RDCs to ordinary refinement by *REFMAC*5. Similarly as for external restraints, the weight given to the NMR restraints relative to the standard geometry restraints needs to be tuned so as to not negatively impact on the original agreement between the model and the X-ray data. Analogously to the *R* factor for X-ray data, the *Q* factor can be calculated to monitor the agreement between NMR data and the model. In principle, a free *Q* factor, with an equivalent meaning to the free *R* factor, could also be defined, but in practice this parameter is never used owing to low levels of redundancy in the NMR data set. For a more detailed description of the refinement protocol, see Rinaldelli *et al.* (2014 ▸).

**Figure 4:**
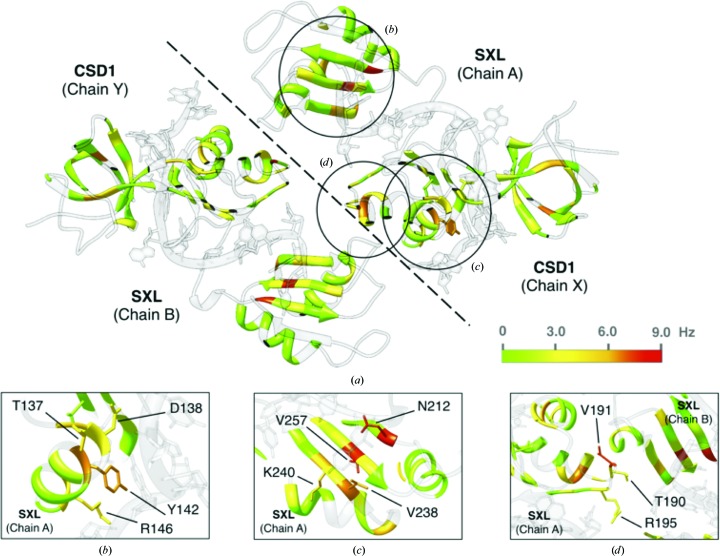
Refinement of the ternary Sxl–Unr–msl2-mRNA regulatory complex. (*a*) The structure refined using the *REFMAC*5 protocol is coloured according to the difference (in absolute value) between experimental RDCs and those back-calculated from the model. No RDCs were measured for residues coloured in white. (*b*, *c*, *d*) Enlarged views for residues reporting the largest differences. Reprinted (adapted) with permission from Carlon *et al.* (2016 ▸). Copyright (2016) American Chemical Society.

Let us consider the refinement of a ternary Sxl–Unr–msl2-mRNA regulatory complex (Carlon, Ravera, Hennig *et al.*, 2016 ▸). This complex consists of both RNA-recognition motifs (RRMs) of Sxl, the first of five cold-shock domains of Unr (CSD1) and an 18-mer single-stranded RNA derived from msl2-mRNA. ‘Diamagnetic’ RDCs were acquired using Pf1 phage-alignment medium on the NH–N and C–N pairs (Hennig *et al.*, 2014 ▸), showing an overall *Q* factor of 0.440 against the original X-ray model (2.8 Å resolution). This value is well beyond the acceptable threshold, as good agreement between RDCs and a structural model is usually indicated by a *Q* factor of 0.2 or below. The refinement was initially performed for the individual RRMs and CSD1 domains, and the presence of putative inter-domain rearrangements was tested through comparison of the alignment tensors obtained from the single structural units. Once the presence of significant rearrangements of such domains was excluded, simultaneous refinement was performed on the overall complex. Retaining values of *R* and *R*_free_ very close to those of the initial model, a decrease in the *Q* factor to 0.144 was observed for RDCs after refinement. Interestingly, the backbone r.m.s.d. between the initial model and that refined using both X-ray and NMR data was less than 0.1. The major modification was observed on the loop of CSD1 in contact with the RNA strand with weaker electron density (Fig. 4 ▸). Therefore, refinement against both diffraction and NMR data could not only confirm highly similar overall protein conformations in the crystal and in solution, but also helped to identify a particular small region that changed its conformation (probably owing to the effects of crystal packing).

In order to exclude the possibility that in-plane or out-of-plane distortions of the NH—N bonds could be the main cause of such improvement (even if within the standard limits), the H atoms were removed from the structure and added back according to ideal geometry using other software, *i.e.* *MolProbity* (Chen *et al.*, 2010 ▸). The recalculated *Q* factor was found to be similar for the structure with the repositioned H atoms, demonstrating that small changes caused by the joint refinement in the position of backbone atoms have a crucial role in determining the position of H atoms and that this, in turn, also has an effect on the agreement with NMR data.

In order to extend the applicability of joint X-ray and NMR structural refinement to higher molecular-weight systems, new optimization functions have recently been implemented in order to improve the reliability of the refinement of multi-domain proteins and protein complexes. A great deal of effort has also been made to pursue the refinement of large symmetric homomultimeric systems. Long-range NMR restraints can be measured on these symmetric assemblies, resulting in a single set of experimental data containing both the structural information of the repeating units and their overall organization within the assembly. For these particular cases, further properties of the tensor related to the specific symmetry of the system can be derived and used as further constraints during the refinement. The new version of *REFMAC*5 containing the mentioned optimization functions will be released soon.

## 6. Conclusions   

The use of prior knowledge in refinement aids the extraction of biologically relevant information from noisy and limited experimental data. Various types of restraints allow the injection of additional information obtained from various sources, ranging from high-resolution structures of related proteins to different experimental observations such as NMR, into the refinement process. However, as experimental data quality degrades, and thus the effective number of observations decreases, it is much easier to achieve good agreement with the prior knowledge, simply because limited data contain negligible information about short-range interactions. Note that good agreement with prior knowledge does not necessarily mean that the derived model corresponds well to the true structure; rather, it only means that the model agrees with the prior knowledge (*i.e.* that the model is chemically/structurally sensible). When considering diffraction data at low resolution, all indicators, as well as the predictive power of the model, must be checked. In other words, when maps are displayed/used as evidence, the part of the atomic model in question (corresponding to the relevant map region) should not be used during the map-calculation procedure; OMIT maps or similar must be used.

The degree of model quality required depends on the questions asked: if questions relate to domain organization, or to the mutual orientation of protein molecules in a complex, then probably low-resolution (3–5 Å) data might provide sufficient evidence. However, if more specific questions are asked that require a higher degree of model accuracy (for example related to specific interatomic distances) then data extending to higher resolution may be needed. In any case, there is always going to be the question as to whether a given model of a crystal is the same as the structure in solution. To answer these concerns, one must perform additional experiments to confirm such hypotheses. One of the techniques that can be used to address such issues involves joint refinement of the model using crystallographic and NMR experimental data such as residual dipolar couplings and pseudocontact shifts. The use of restraints derived from NMR data can not only improve macromolecular crystallographic model refinement but also identify discrepancies between the structure in the crystal and in solution.

In general, we believe that all available sources of experimental and theoretical information relevant to the molecule under refinement should be utilized in order to deliver the best possible structural model for the current state of the molecule. It is hoped that this will lead to the acquisition of more accurate and reliable models that are suitable for addressing important biological questions.

## Supplementary Material

Supplementary Table 1.. DOI: 10.1107/S2059798318000979/ba5283sup1.pdf

## Figures and Tables

**Table 1:** Refinement and geometry statistics corresponding to the model with PDB code 1jkt before and after automated refinement using the *LORESTR* pipeline

	*R*_work_ (%)	*R*_free_ (%)	Ramachandran outliers (%)	Ramachandran favoured (%)	Clashscore percentile	*MolProbity* percentile
Initial	24.3	28.4	16.2	61.1	3.2	4.6
Final	16.9	20.8	2.6	93.4	73.0	66.1
