# Supplementary material for: Overview of refinement procedures within REFMAC5: utilizing data from different sources
Source: Acta Crystallogr D Struct Biol. 2018 Mar 2;74(Pt 3):215–27. doi: 10.1107/S2059798318000979 (PMC5947762; doi:10.1107/S2059798318000979)
Supplement: Supplementary file 1 [file d-74-00215-sup1.pdf]

Acta Cryst D STRUCTURAL BIOLOGY

Volume 74 (2018)

Supporting information for article:

Overview of refinement procedures within *REFMAC*5: utilising data from different sources

Oleg Kovalevskiy, Robert A. Nicholls, Fei Long, Azzurra Carlon and Garib N. Murshudov

PDB code (first)	Starting R_{free}	Starting MolProbity score percentile	R_{free} , reciprocal refinement	MolProbity score*, reciprocal refinement	R_{free} , reciprocal refinement + cleaning[+]	MolProbity score*, reciprocal refinement + cleaning[+]	PDB code (second)	Starting R_{free}	Starting MolProbity score percentile	R_{free} , reciprocal refinement	MolProbity score*, reciprocal refinement	R_{free} , reciprocal refinement + cleaning[+]	MolProbity score*, reciprocal refinement + cleaning[+]
3lrb	0.322	4.5	0.291	29.7	0.288	30.3	3l1l	0.281	23.1	0.27	35.3	0.272	37.5
3syn	0.235	39.7	0.24	51.8	0.238	53.3	2px3	0.241	20.8	0.258	39.2	0.259	38.7
2det	0.31	2.4	0.282	5.7	0.275	12.5	2der	0.266	10.4	0.256	16.2	0.255	19.7
1qzx	0.422	13	0.348	38.1	0.348	38.1	3kl4	0.314	22.4	0.353	36.7	0.36	37.4
2r65	0.338	10.4	0.31	25.6	0.316	33.2	2r62	0.35	2.4	0.355	3.9	0.353	3.3
1ixs	0.287	21.8	0.249	32.9	0.251	34	1hqc	0.251	12	0.28	25.4	0.281	23.9
1piw	0.266	22.1	0.245	48	0.237	49.2	1ps0	0.296	21.5	0.267	42.1	0.263	52.9
1f9k	0.237	57.3	0.224	71	0.222	75.3	1fay	0.237	68.8	0.225	80.7	0.226	84.4
3l1l	0.281	23.1	0.27	35.3	0.272	37.5	3lrb	0.322	4.5	0.291	29.7	0.288	30.3
2px3	0.241	20.8	0.258	39.2	0.259	38.7	3syn	0.235	39.7	0.24	51.8	0.238	53.3
3u6n	0.299	67.1	0.291	73.1	0.288	73.1	3naf	0.303	37.7	0.281	38.6	0.286	49.3
3qa8	0.356	11.3	0.361	25.1	0.365	25.8	3rzf	0.365	22.2	0.319	34.8	0.319	36.2
2ec8	0.263	12.6	0.284	22.6	0.277	17.5	2e9w	0.27	23.6	0.249	45.6	0.249	45.6
1xd4	0.341	77.4	0.263	81.5	0.249	77.8	1xdv	0.405	54.2	0.299	63.9	0.284	67.1
3mkt	0.351	0.3	0.361	4	0.356	4	3mku	0.357	1.3	0.37	29.1	0.37	29.1
2deu	0.341	6.6	0.307	16.4	0.303	20.5	2der	0.266	10.4	0.257	15.4	0.254	14.4
3naf	0.303	37.7	0.281	39.5	0.28	38.5	3u6n	0.299	67.1	0.291	73.1	0.292	74.2
3aql	0.253	6.4	0.252	11.2	0.248	11	3aqk	0.281	9.1	0.247	37.2	0.265	59.1
4a2p	0.291	65.8	0.269	61.9	0.272	66.1	4a36	0.266	59.3	0.259	59.3	0.253	62.3
1g5g	0.271	10.9	0.251	46	0.252	45.1	3maw	0.297	92.5	0.301	79.7	0.307	91.7
1hqc	0.251	12	0.277	24.8	0.274	20.7	1ixr	0.365	2.1	0.33	14.1	0.33	14.1
1qzw	0.349	0.6	0.262	31	0.269	20	3kl4	0.314	22.4	0.347	32.1	0.347	35.8
2qkw	0.373	56.2	0.309	30.6	0.309	32.3	3hgk	0.342	29.9	0.355	50.5	0.331	46.8
3kl4	0.314	22.4	0.347	32.1	0.347	35.8	1qzw	0.349	0.6	0.277	20	0.269	20
1rh5	0.299	21.2	0.285	36.8	0.281	39.9	1rhz	0.347	5.4	0.333	29.1	0.327	30.8
3k3w	0.306	50.7	0.268	75	0.229	62.9	3ml0	0.28	88.8	0.23	80.7	0.209	73.3
3uj0	0.306	66.2	0.301	68.4	0.301	73	3t8s	0.296	61.3	0.286	76.4	0.286	76.9
3mt5	0.27	65.8	0.247	60	0.247	60	3naf	0.303	37.7	0.269	34.5	0.265	34.4

* - Molprobity score percentile

[+]- by cleaning we mean applying procedure for removing residues with improper setereochemistry, high B-factors, etc. (see the text)
